# Association between Hemoglobin Glycation Index and NAFLD in Chinese Nondiabetic Individuals

**DOI:** 10.1155/2019/8748459

**Published:** 2019-12-19

**Authors:** Di-Shuang Hu, Sheng-Hao Zhu, Xu Li, Qin-Fen Chen, Chun-Jing Lin, Dan-Hong Fang, Jian-Sheng Wu

**Affiliations:** ^1^Department of Gastroenterology, The First Affiliated Hospital of Wenzhou Medical University, Wenzhou 325000, Zhejiang, China; ^2^Wenzhou Medical University RenJi College, Wenzhou 325000, Zhejiang, China; ^3^Medical and Health Care Center, The First Affiliated Hospital of Wenzhou Medical University, Wenzhou 325000, Zhejiang, China

## Abstract

**Purpose:**

Limited studies have preliminarily identified a positive association between nonalcoholic fatty liver disease (NAFLD) and hemoglobin glycation index (HGI). However, this association has not been fully established. We aim to investigate the association between NAFLD and HGI in Chinese nondiabetic individuals and to construct a risk score based on HGI to predict a person's risk of NAFLD.

**Methods:**

After strict exclusion criteria, 5,903 individuals were included in this retrospective cross-sectional study. We randomly selected 1,967 subjects in the enrollment to obtain an equation of linear regression, which was used to calculate predicted HbA1c and drive HGI. The other subjects were classified into four categories according to HGI level (≤−0.22, −0.21∼0.02, 0.03∼0.28, and ≥0.29). All subjects retrospectively reviewed the baseline characteristics, laboratory examinations, and abdominal ultrasonography.

**Results:**

The prevalence of NAFLD in this population was 20.7%, which increases along with the growth of HGI levels (*P* < 0.001). Adjusted to multiple factors, this trend still remained significant (OR: 1.172 (95% CI, 1.074–1.279)). The combined NAFLD risk score based on HGI resulted in an area under the receiver operator characteristic curve (AUROC) of 0.85 provided sensitivity, specificity, positive predictive value, and a negative predictive value for NAFLD of 84.4%, 71.3%, 65.0%, and 88.0%, respectively.

**Conclusions:**

NAFLD is independently associated with HGI levels in Chinese nondiabetic individuals. And, NAFLD risk score may be used as one of the risk predictors of NAFLD in nondiabetic population.

## 1. Introduction

Nonalcoholic fatty liver disease (NAFLD) includes a wide spectrum of progressive liver disorders ranging from simple steatosis to nonalcoholic steatohepatitis (NASH), which may lead to liver cirrhosis and even hepatocellular carcinoma [1–3]. NAFLD has been proved to be associated with insulin resistance-related diseases, such as type 2 diabetes, obesity, and metabolic syndrome, and induced atherosclerosis [4–7]. The global prevalence of NAFLD is currently estimated to be 14–32% [6]. And, in China, the incidence of NAFLD is reckoned to be approximately 20.0% [8], which is rapidly increasing due to the more frequent occurrence of metabolic syndrome [9]. Currently, NAFLD has become the main cause of abnormal liver biochemical indicators in health checkups in China, which is the major issue of chronic liver disease worldwide.

Glycated hemoglobin (HbA1c) represents a 2-3- month average of blood glucose concentration [10], which is the gold standard for evaluating glycemic status and the efficacy of various therapeutic schedules [11, 12]. However, recent researches had shown that considerable biological variation of HbA1c was not only affected by blood glucose levels but also influenced by interindividual biological differences [13, 14]. That means, even at the same blood glucose level, the level of HbA1c could be different. To overcome these disadvantages, in 2002, Hempe et al. developed a mathematical method which termed hemoglobin glycation index (HGI) to quantify the disparity between measured HbA1c and predicted HbA1c [15, 16]. Nowadays, a few articles have been reported studying the relationship between HGI and nondiabetic individuals [17–19]. Obviously, HGI has been used to represent the nonenzymatic protein glycation, which is known to play a key role in the pathogenesis of NAFLD [20, 21]. However, only two studies had been carried out to assess the correlation between HGI and NAFLD in nondiabetic individuals [17, 22]. The aims of our study were (1) to assess the prevalence of NAFLD among nondiabetic Chinese adults; (2) to investigate whether HGI is associated with the prevalence of NAFLD and related to biomarkers in Chinese nondiabetic subjects; and (3) to construct a risk score to conveniently predict a person's risk of NAFLD.

## 2. Materials and Methods

### 2.1. Subjects

**Figure 1:**
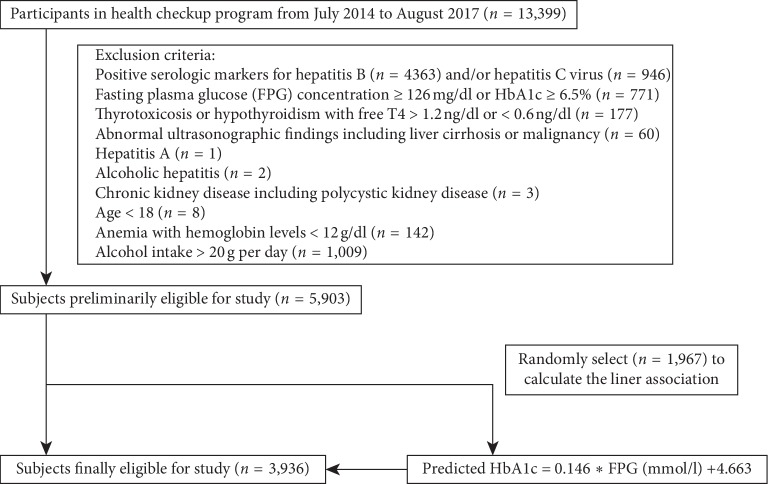
Flowchart of subjects across the study.

This cross-sectional analysis was performed in the First Affiliated Hospital of Wenzhou Medical University (Zhejiang, China) from July 2014 to August 2017. Initial data were obtained from 13,399 subjects volunteered for a comprehensive health checkup. Finally, 5,903 subjects were excluded from the initial study population according to the following exclusion criteria: (1) positive serologic markers for hepatitis B (*n* = 4363) and/or hepatitis C (*n* = 946) virus; (2) fasting plasma glucose (FPG) concentration ≥126 mg/dl or HbA1c ≥6.5% (*n* = 771); (3) thyrotoxicosis or hypothyroidism with free *T*4 > 1.2 ng/dl or <0.6 ng/dl (*n* = 177); (4) abnormal ultrasonographic findings including liver cirrhosis or malignancy (*n* =60), hepatitis A (*n* = 1), alcoholic hepatitis (*n* = 2), chronic kidney disease including polycystic kidney disease (*n* = 3); (5) age <18 (*n* = 8); (6) anemia with hemoglobin levels <12 g/dl (*n* = 142); (7) alcohol intake >20 g per day (*n* = 1009). After applying the above exclusion criteria, the total number of subjects eligible for the study was 5,903 (3,546 male and 2,357 female) (Figure 1).

### 2.2. Measurements and Laboratory Tests

Patients' heights (measured to the nearest 0.1 cm) and weights (measured to the nearest 0.1 kg) were measured with light clothing by well-trained nurses in the morning. Body mass index (BMI, kg/m^2^) was calculated as weight divided by the height. Blood pressure was measured in the right arm at the same level as the left atrium in a seated state with a standard automatic sphygmomanometer (Omron, model 705 cp, Kyoto, Japan). After 12 h of overnight fasting, blood samples were collected from the antecubital vein by experienced nurses. Blood routine including hemoglobin, platelets, white blood cells (WBC), and biochemical markers such as albumin, HbA1c, FPG, total cholesterol (TC), low-density lipoprotein cholesterol (LDL-C), high-density lipoprotein cholesterol (HDL-C), triglyceride (TG), gamma glutamyltransferase (GGT), alanine aminotransferase (ALT), and aspartate aminotransferase (AST) were subsequently analyzed by an automated analyzer (Abbott AxSYM, Park, IL). All the participants routinely underwent the abdominal ultrasonography scanning (Siemens, Munich, Germany) by experienced radiologists, all of whom were blinded to the clinical status of the subjects. And, the fatty liver was diagnosed based on the guidelines for the diagnosis and treatment of nonalcoholic fatty liver disease, including (1) the near field echo of the liver is diffusely enhanced and stronger than the echo of the kidney; (2) liver brightness; and (3) vascular blurring [23].

### 2.3. Calculation of HGI, HSI, and NFS

We randomly selected 1,967 subjects in the enrollment, and by inserting FPG into a population regression equation expressing the linear association between HbA1c and FPG; we drove the predicted HbA1c, which is defined as follows: HbA1c=0.146*∗*FPG(mmol/1)+4.663. And, HGI is expressed as measured HbA1c minus predicted HbA1c. In a word, HGI is calculated as the difference between the observed HbA1c and the predicted value of HbA1c based on plasma glucose concentration [15, 24]. The other 3,936 subjects were classified into four categories according to HGI levels (≤−0.22, −0.21∼0.02, 0.03∼0.28, and ≥0.29), which were defined as lowest, low, moderate, and high, respectively.

The Hepatic Steatosis Index (HSI) is a preliminary screening tool for NAFLD, which uses a formula based on BMI, ALT (IU/L), and AST (IU/L), and the presence or absence of diabetes as follows: HSI=8*∗*(ALT/AST ratio)+BMI(+2, if T2DM; +2, if female). Additionally, NAFLD was defined as HSI above 36 [25].

The NAFLD Fibrosis Score (NFS) is an invasive approach to detect liver fibrosis as follows: NFS=−1.675+0.037*∗*age(years)+0.094*∗*BMI(kg/m^2^)+1.13*∗*impaired fasting glucose (IFG)  or  diabetes  (yes=1, no=0)+0.99*∗*AST/ALT  ratio–0.013*∗*platelet(*∗*10^9^/L)–0.66*∗*albumin(g/dL). NFS <−1.455 predicts a low probability of advanced fibrosis [26].

### 2.4. Statistical Analyses

Data for continuous variables were expressed as mean values with standard deviation and as percentages for categorical variables. One-way analysis of variance with Bonferroni's method, post hoc analysis, and Chi-squared test were used to compare statistical differences in the characteristics of the study participants at baseline among the groups. Univariate logistic regression analysis was acted out to explore the risk factors of NAFLD by ultrasonography. Multiple logistic regression analysis was executed to estimate the OR and 95% CI for the association of categorized HGI levels with the risk of NAFLD. The area under the curve of the receiving operating characteristic (ROC) was used to evaluate the sensitivity, specificity, positive predictive value (PPV), and negative predictive value (NPV) of the proposed risk score for the prediction of NAFLD. Statistical data analysis was performed by using the SPSS version 25.0 for Windows. And, statistical significance was set at <0.05.

## 3. Results

### 3.1. Characteristics of Study Participants According to HGI Quartiles

**Figure 2:**
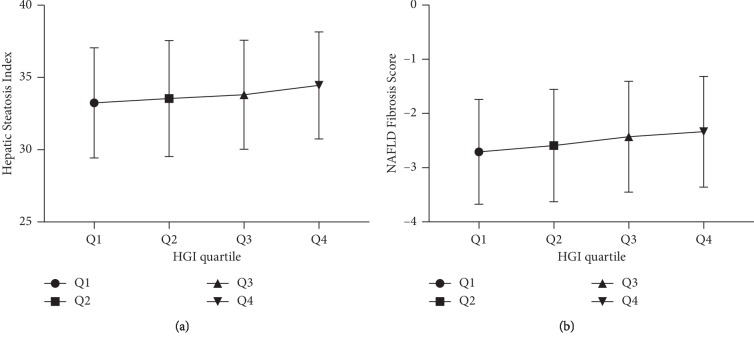
A relationship between HGI and HSI (a) as well as HGI and NFS (b).

**Figure 3:**
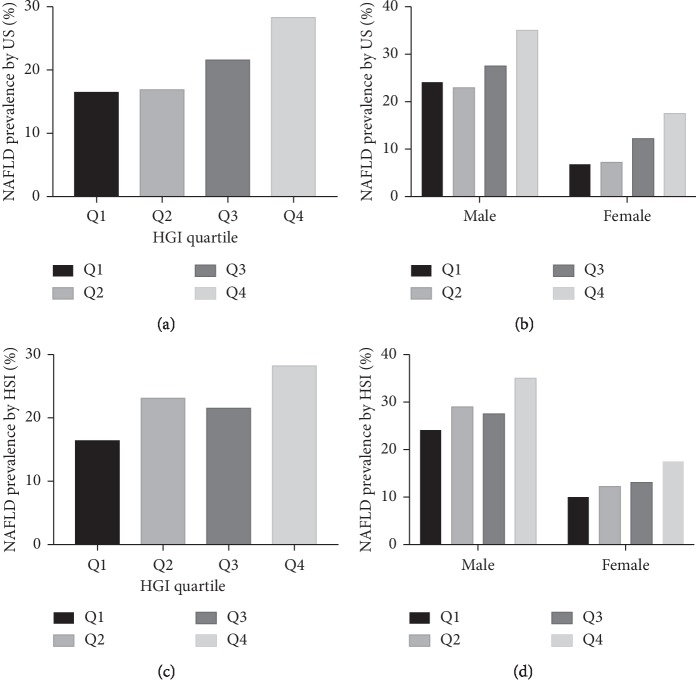
The proportion of subjects with NAFLD by HGI category within the nondiabetic range by US (a, b) and HSI (c, d). *P* < 0.001 by *χ*^2^ test for trend.

Study population comprised 3,936 participants, of whom 2,349 (59.7%) were male. Of the subjects at baseline, the participants older than 50 years old and with a BMI ≥25 were 1,437 (36.5%) and 1,278 (32.5%), respectively. Overall, the participants were relatively young and not obese, with the mean age of 47.7 ± 11.0 years old and mean BMI 23.8 ± 3.2 kg/m^2^. The baseline of anthropometric features and biochemical findings of the study population was stratified according to quartiles of HGI value showed in Table 1. Statistically significant differences among groups were found with respect to the following variables: (1) the anthropometric parameters such as age, gender, SBP, DBP, and BMI; (2) the inflammatory markers such as platelets and WBC; (3) the biomarker of liver damage, e.g., ALT, AST, and GGT; (4) the biochemical indicators such as FPG, creatinine, TC, TG, HDL-C, and LDL-C; (5) the indicator of hepatic steatosis such as HSI; (6) the indicator of hepatic fibrosis, NFS, for example, see Figure 2. Accordingly, we found an increase in the prevalence of hepatic steatosis diagnosed by ultrasonography and HSI in Q1∼Q4 groups (Figure 3).

### 3.2. Association between HGI and the Prevalence of NAFLD

To investigate the potential interactions affecting the prevalence of NAFLD, univariate logistic regression analysis was performed as shown in Table 2. It was shown that male, fat, hyperlipidemia, high levels of HbA1c, ALT, AST, GGT, and HSI, and the inflammatory markers were risk factors for NAFLD. Furthermore, in our study, HGI was found having a strong link with the incidence of NAFLD.  Table 3 showed the independent association between NAFLD defined by abdominal ultrasound and HGI level performed by multivariate analysis. Before adjustment for any multiple confounding factors, the high HGI quartiles were associated with higher risk of hepatic steatosis (OR: 1.281 (95% CI, 1.194–1.375)). After adjustment for age, gender, BMI, ALT, AST, HDL-C, TG, FPG, GGT, WBC, blood pressure, alcohol, smoking, and albumin, this trend still remained significant (OR: 1.172 (95% CI, 1.074–1.279)).

### 3.3. Construction of a Risk Score for NAFLD

**Figure 4:**
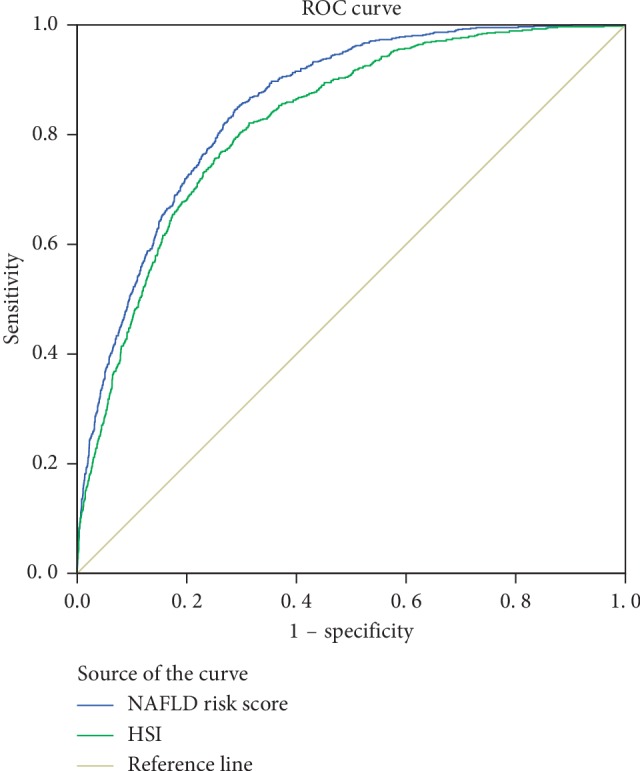
Receiver operating characteristics curves of NAFLD risk score and HSI for diagnosing the risk of NAFLD. AUC, area under the curve; HSI, hepatic steatosis index.

According to the results, the variables such as HGI, TG, FPG, BMI, ALT, HDL-C, and WBC were the key risk factors (*P* < 0.05). We put them into the model, which determined the risk of NAFLD (NAFLD risk score=0.363 HGI+0.744 ln TG+1.674 ln FPG+0.213 BMI+1.361 ln ALT − 0.425 HDL − C+0.130 WBC − 14.381). The area below the ROC curve of this model was 0.85, and an optimal cutoff value of 0.174 generated sensitivity, specificity, positive predictive value, and a negative predictive value for NAFLD of 84.4%, 71.3%, 65.0%, and 88.0%, respectively. The AUC of HSI was 0.82, and a cutoff value of 33.1 gave a sensitivity of 82.1% and a specificity of 68.6% for detecting the presence of NAFLD (Figure 4).

## 4. Discussion

Previous studies have reported that HGI represents the degree of nonenzymatic hemoglobin glycation, and it has been found to promote the development of microvascular and macrovascular complications in diabetic subjects. Using HGI analysis to the Diabetes Control and Complications Trial (DCCT), it has been proved that the higher the HGI, the greater the risk of retinopathy and nephropathy in the patients with type 1 diabetes [27]. Similarly, in population with type 2 diabetes participating to the Action to Control Cardiovascular Risk in Diabetes (ACCORD) trial, we could get the same conclusion at the baseline [28]. Furthermore, recent studies had shown that even within the normal blood glucose level, the higher HGI level may identify subjects with an increased risk of insulin resistance, carotid atherosclerosis [18], and fatty liver [17, 29].

The first study demonstrated the relationship between NAFLD and HGI in 1,120 white individuals without diabetes was in 2017. When adjusted for age, gender, and BMI, individuals in the highest quartile of HGI exhibited a 1.6-fold increased odd of having hepatic steatosis compared with subjects in the lowest. Another one published recently of 14,465 nondiabetic, Korea subjects showed that a high HGI was associated with a 1.56-fold increase in the risk of hepatic steatosis after adjusting more factors than the previous one. However, they did not further construct a risk score based on HGI to predict a person's risk of NAFLD, which can be useful for the clinician.

Our study demonstrated that HGI was one of the independent risk factors for incident NAFLD among nondiabetic Chinese medical examination population. The result that 20.7% of the nondiabetic population who was affected by NAFLD was equivalent to the ultrasound results of one in every four adults with NAFLD in China [30]. In comparison with subjects with low HGI, those with higher HGI were likely to be male, old, and obese, having abnormal blood lipid profile, increased levels of liver enzyme, HSI, inflammatory biomarkers, and more susceptible to NAFLD. Moreover, in Figure 2, we could intuitively discover that elevated levels of HGI can promote hepatic steatosis and fibrosis. In addition, we further constructed a NAFLD risk score based on HGI, BMI, WBC, TG, ALT, FPG, and HDL-C for diagnosing the risk of NAFLD, which showed a more valuable performance to identify NAFLD than HSI according to a ROC analysis (Figure 4). As far as we know, this is the largest cross-sectional study in this research area about Chinese.

Nowadays, not only the association between HGI and NAFLD has not been fully evaluated but also NAFLD pathogenesis is still unclear, so the pathophysiological mechanisms involved in the associations between each other are still undefined. Through this retrospective cross-sectional study, there are three suggested mechanisms to explain the association between HGI and NAFLD. First, we found a positive association between HGI and inflammatory biomarkers including WBC and platelet count. Obviously, chronic inflammation plays a key role in the progress of NAFLD, and insulin resistance is thought to be a core component of NAFLD. Impaired mitochondria can result in incomplete fat oxidation and generation of toxic lipid intermediates, which generate a large amount of reactive oxygen species (ROS) and reactive nitrogen free radicals (RNS) and lead to inflammation. Then, inflammation may impair insulin signaling and exacerbate liver fatty infiltration, even fuel the transition from NAFLD to NASH, and liver cirrhosis, even hepatocellular carcinoma [29, 31, 32]. Furthermore, study had proved that methods reducing adipose tissue inflammation may improve insulin resistance in NAFLD [33]. Accordingly, in our study, individuals with high HGI showed increased levels of inflammatory biomarkers such as platelets and WBC counted independently of other confounding factors. Second, AGEs are stable irreversible polymerizations of various macromolecules (proteins, lipids, nucleic acids, etc.) and spontaneously reacted with reducing monosaccharides under nonenzymatic conditions, which alter the instruction and function of proteins. And, the dose of hepatic AGEs matches with the AGEs of dietary [34]. Studies had shown that AGEs generally combined with activation of RAGE downstream pathway, and AGE/RAGE triggered further inflammation, oxidative stress, and impair insulin signaling and thus exacerbated the development and progression of NAFLD [35]. Felipe et al. had found that the level of AGEs in tissues can be reflected by skin intrinsic fluorescence (SIF) markers, and the increase of SIF concentration is associated with HGI [36]. Third, obesity and the consequent adipose metabolic dysfunction represent important risk factors for the development and progression of NAFLD, and weight loss can improve lipid metabolism in the liver [37]. Obesity may lead to an imbalanced production of pro- and anti-inflammatory adipokines secreted from adipose tissue, which contributes to the pathogenesis of NAFLD. Correspondingly, we found HGI levels were positively associated with measures of BMI.

Our study had some limitations that should be taken into account. First, our study subjects were derived from a single center; for each population, new regression models should be derived, and multicentered research should be performed to further confirm the association in the next step. Second, our biochemical parameters, such as plasma glucose and HbA1c, were measured once. Even though this approach is commonly used in clinical research, between-individual variability of glucose homeostasis parameters may have led to some imprecisions in the stratification of study population into HGI quartiles. Third, the diagnosis of liver steatosis was performed by ultrasound scanning rather than by invasive methods such as liver biopsy or expensive noninvasive approaches such as proton magnetic resonance spectroscopy or computed tomographic scanning. However, ultrasonography is the most commonly used method to diagnose hepatic steatosis in clinical practice and epidemiological studies. Although liver biopsy is a standard criterion for NAFLD diagnosis, the diagnosis of fatty liver was based on ultrasound imaging with 90% sensitivity and 80% specificity [38].

## 5. Conclusion

In conclusion, we further confirmed that NAFLD had an association with HGI level in nondiabetic individuals, and these associations were independent of obesity and other metabolic components. And, NAFLD risk score could be used as one of the risk predictors of NAFLD in nondiabetic population. But the causal relationship between HGI level and NAFLD in nondiabetic individuals is not undefined, while further perspective study should be taken.

## Figures and Tables

**Table 1:** Baseline characteristics of study participants according to the HGI.

Variables		Total *n* = 3936	Quartile 1 (≤−0.22) *n* = 998	Quartile 2 (−0.21∼0.02) *n* = 985	Quartile 3 (0.03∼0.28) *n* = 975	Quartile 4 (≥0.29) *n* = 978	*F* or *χ*^2^
Gender (male/female)		1587/2349	443/555	388/597	378/597	378/600	9.33^*∗∗∗*^
Age (years)		47.74 ± 11.01	42.81 ± 9.46	46.29 ± 10.59^a^	48.95 ± 10.96^ab^	53.01 ± 10.41^abc^	170.37^*∗*^
SBP (mmHg)		123.69 ± 18.56	120.85 ± 17.43	122.32 ± 17.89	124.91 ± 19.24^ab^	126.74 ± 19.12^ab^	20.09^*∗*^
DBP (mmHg)		73.12 ± 12.63	71.91 ± 12.49	72.86 ± 12.63	73.52 ± 12.78^a^	74.22 ± 12.5^ab^	6.02^*∗*^
BMI (kg/m^2^)		23.76 ± 3.16	23.17 ± 3.08	23.54 ± 3.21^a^	23.91 ± 3.12^ab^	24.43 ± 3.08^abc^	29.34^*∗*^
WBC (×10^9^/L)		6.04 ± 1.61	5.79 ± 1.53	6.02 ± 1.55^a^	6.07 ± 1.66^a^	6.30 ± 1.67^abc^	16.88^*∗*^
HB (g/L)		145.19 ± 14.90	146.44 ± 15.21	145.97 ± 15.07	144.69 ± 14.64^a^	143.64 ± 14.51^ab^	7.15^*∗*^
PLT (×10^9^/l)		231.71 ± 53.25	226.23 ± 51.44	232.01 ± 53.57	231.61 ± 51.16	237.07 ± 56.23^a^	6.86^*∗*^
ALB (g/L)		44.93 ± 3.05	45.52 ± 3.07	45.16 ± 3.03^a^	44.59 ± 3.07^ab^	44.44 ± 2.89^ab^	27.48^*∗*^
ALT (U/L)		27.68 ± 33.13	26.09 ± 20.5	26.05 ± 18.57	26.81 ± 19.29	31.82 ± 57^ab^	6.91^*∗*^
AST (U/L)		27.28 ± 16.74	25.16 ± 14.01	25.17 ± 10.37	26.07 ± 9.81	28.74 ± 26.75^abc^	10.09^*∗*^
GGT (U/L)		44.94 ± 66.78	42.46 ± 88.12	42.72 ± 62.15	44.37 ± 51.89	50.27 ± 58.5^ab^	2.92^*∗∗∗*^
FPG (mmol/L)		4.67 ± 0.61	4.65 ± 0.53	4.63 ± 0.55	4.64 ± 0.62	4.76 ± 0.72^abc^	8.92^*∗*^
Creatinine (umol/L)		67.55 ± 14.15	66.16 ± 13.73	67.58 ± 14.84^a^	68.23 ± 14.12^a^	68.25 ± 13.83^a^	4.76^*∗∗*^
TC (mmol/L)		5.29 ± 1.05	5.09 ± 0.97	5.26 ± 1.01^a^	5.35 ± 1.04^a^	5.46 ± 1.13^ab^	23.39^*∗*^
TG (mmol/L)		1.74 ± 1.23	1.62 ± 1.21	1.70 ± 1.21	1.74 ± 1.23^a^	1.93 ± 1.26^abc^	11.05^*∗*^
HDL-C (mmol/L)		1.28 ± 0.33	1.32 ± 0.34	1.29 ± 0.33	1.29 ± 0.33	1.24 ± 0.31^abc^	9.93^*∗*^
LDL-C (mmol/L)		3.16 ± 0.85	3.00 ± 0.76	3.13 ± 0.81^a^	3.22 ± 0.86^a^	3.31 ± 0.92^ab^	24.29^*∗*^
HSI		32.49 ± 4.84	31.89 ± 4.76	32.22 ± 5.04^a^	32.50 ± 4.75^ab^	33.36 ± 4.70^ab^	27.62^*∗*^
NFS		−2.52 ± 1.02	−2.71 ± 0.97	−2.60 ± 1.04	−2.42 ± 1.02^a^	−2.34 ± 1.02^abc^	17.00^*∗*^
NAFLD (%)
Hepatic US		815 (20.7)	164 (16.4)	165 (16.8)	210 (21.5)	276 (28.2)	54.53^*∗*^
HSI		875 (22.2)	174 (17.4)	221 (22.4)	207 (21.2)	273 (27.9)	32.14^*∗*^
HbA1c (%)		5.38 ± 0.40	4.89 ± 0.22	5.25 ± 0.11^a^	5.50 ± 0.12^ab^	5.88 ± 0.22^abc^	5599.99^*∗*^

^*∗*^ *P* < 0.001, ^*∗∗*^*P* < 0.01, ^*∗∗∗*^*P* < 0.05; ^a^*P* < 0.05 compared with quartile 1, ^*b*^*P* < 0.05 compared with quartile 2, ^*c*^*P* < 0.05 compared with quartile 3. Data are presented as mean ± SD or median (interquartile) or *n* (%); SBP, systolic blood pressure; DBP, diastolic blood pressure; BMI, body mass index; WBC, white blood cell; HB, hemoglobin; PLT, blood platelet; ALB, albumin; ALT, alanine aminotransferase; AST, aspartate aminotransferase; GGT, gamma glutamyltransferase; TC, total cholesterol; TG, triglyceride; HDL-C, high-density lipoprotein cholesterol; LDL-C, low-density lipoprotein cholesterol; HSI, Hepatic Steatosis Index; NFS, NAFLD Fibrosis Score; NAFLD, nonalcoholic fatty liver disease; US, ultrasonography; HbA1c, glycosylated hemoglobin; HGI, hemoglobin glycation index.

**Table 2:** Univariate logistic regression analysis for the risk of NAFLD by ultrasonography.

Parameters	Odds ratio	Lower 95% CI	Upper 95% CI	*P*
Gender (male/female)	3.155	2.627	3.789	<0.001
Age (years)	1.004	0.997	1.011	0.230
BMI (kg/m^2^)	1.406	1.364	1.451	<0.001
Hypertension (%)	1.801	1.514	2.413	<0.001
Alcohol intake	1.627	1.392	1.901	<0.001
Smoking	1.527	1.292	1.804	<0.001
FPG (mmol/L)	1.716	1.509	1.952	<0.001
TG (mmol/L)	1.837	1.709	1.975	<0.001
TC (mmol/L)	1.307	1.216	1.404	<0.001
HDL-C (mmol/L)	0.131	0.097	0.176	<0.001
LDL-C (mmol/L)	1.357	1.241	1.484	<0.001
ALT (U/L)	1.037	1.032	1.041	<0.001
AST (U/L)	1.038	1.031	1.045	<0.001
GGT (U/L)	1.009	1.007	1.010	<0.001
WBC (×10^9^/L)	1.300	1.241	1.302	<0.001
HB (g/L)	1.047	1.041	1.053	<0.001
PLT (×10^9^/l)	1.002	1.001	1.004	<0.001
ALB (g/L)	1.116	1.087	1.145	<0.001
HGI	2.196	1.787	2.698	<0.001

BMI, body mass index; FPG, fasting blood glucose; TC, total cholesterol; TG, triglyceride; HDL-C, high-density lipoprotein cholesterol; LDL-C, low-density lipoprotein cholesterol; ALT, alanine aminotransferase; AST, aspartate aminotransferase; GGT, gamma glutamyltransferase; WBC, white blood cell; HB, hemoglobin; PLT, blood platelet; ALB, albumin; HGI, hemoglobin glycation index.

**Table 3:** Risk of NAFLD by ultrasonography according to HGI quartile and other variables.

Variables	Odds ratio (95% CI)			
	Model 1	Model 2	Model 3	Model 4
HGI	1.281 (1.194∼1.375)	1.282 (1.189–1.384)	1.284 (1.184∼1.392)	1.172 (1.074∼1.279)
Gender		3.114 (2.591∼3.743)	2.316 (1.845∼2.906)	1.315 (1.018∼1.699)
Age (years)		0.997 (0.990∼1.005)	0.993 (0.984∼1.002)	1.005 (0.995∼1.015)
TG (mmol/L)			1.562 (1.449∼1.684)	1.226 (1.131∼1.331)
Hypertension			1.396 (1.147∼1.700)	1.042 (0.842∼1.288)
Alcohol			0.840 (0.691∼1.021)	0.831 (0.673∼1.026)
Smoking			0.817 (0.665∼1.004)	0.759 (0.606∼0.951)
Alb (g/L)			1.061 (1.029∼1.095)	1.110 (1.071∼1.149)
FPG (mmol/L)			1.437 (1.247∼1.657)	1.326 (1.138∼1.544)
GGT (U/L)			1.003 (1.001∼1.004)	1.002 (1.001∼1.004)
BMI (kg/m^2^)				1.284 (1.240∼1.329)
AST (U/L)				0.987 (0.973∼1.000)
HDL-C (mmol/L)				0.385 (0.262∼0.568)
ALT (U/L)				1.021 (1.013∼1.029)
WBC (×10^9^/L)				1.162 (1.098∼1.231)

Model 1: no adjustment; model 2: adjusted for age and sex; model 3: model 2 + TG, hypertension, alcohol, smoking, albumin, FPG, and GGT; model 4: model 3 + BMI, AST, ALT, HDL-C, and WBC. TG, triglyceride; FPG, fasting blood glucose; GGT, gamma glutamyltransferase; BMI, body mass index; AST, aspartate aminotransferase; HDL-C, high-density lipoprotein cholesterol; ALT, alanine aminotransferase; WBC, white blood cell; Alb, albumin; NAFLD, nonalcoholic fatty liver disease; HGI, hemoglobin glycation index.

## Data Availability

The data used to support the findings of this study are available from the corresponding author upon request.
